# Circadian rhythms in mitochondrial respiration

**DOI:** 10.1530/JME-17-0196

**Published:** 2018-01-29

**Authors:** Paul de Goede, Jakob Wefers, Eline Constance Brombacher, Patrick Schrauwen, Andries Kalsbeek

**Affiliations:** 1 Department of Clinical ChemistryLaboratory of Endocrinology, Academic Medical Center (AMC), University of Amsterdam, Amsterdam, The Netherlands; 2 Department of Human Biology and Human Movement SciencesMaastricht University Medical Center (MUMC), Maastricht, The Netherlands; 3 Department of Endocrinology and MetabolismAcademic Medical Center (AMC), University of Amsterdam, Amsterdam, The Netherlands; 4 Hypothalamic Integration Mechanisms GroupNetherlands Institute for Neuroscience (NIN), Amsterdam, The Netherlands

**Keywords:** mitochondria, mitochondrial functioning, circadian clock, circadian rhythm, respiration

## Abstract

Many physiological processes are regulated with a 24-h periodicity to anticipate the environmental changes of daytime to nighttime and vice versa. These 24-h regulations, commonly termed circadian rhythms, among others control the sleep–wake cycle, locomotor activity and preparation for food availability during the active phase (daytime for humans and nighttime for nocturnal animals). Disturbing circadian rhythms at the organ or whole-body level by social jetlag or shift work, increases the risk to develop chronic metabolic diseases such as type 2 diabetes mellitus. The molecular basis of this risk is a topic of increasing interest. Mitochondria are essential organelles that produce the majority of energy in eukaryotes by converting lipids and carbohydrates into ATP through oxidative phosphorylation. To adapt to the ever-changing environment, mitochondria are highly dynamic in form and function and a loss of this flexibility is linked to metabolic diseases. Interestingly, recent studies have indicated that changes in mitochondrial morphology (i.e., fusion and fission) as well as generation of new mitochondria are dependent on a viable circadian clock. In addition, fission and fusion processes display diurnal changes that are aligned to the light/darkness cycle. Besides morphological changes, mitochondrial respiration also displays diurnal changes. Disturbing the molecular clock in animal models leads to abrogated mitochondrial rhythmicity and altered respiration. Moreover, mitochondrial-dependent production of reactive oxygen species, which plays a role in cellular signaling, has also been linked to the circadian clock. In this review, we will summarize recent advances in the study of circadian rhythms of mitochondria and how this is linked to the molecular circadian clock.

## Introduction

Due to the continuous rotation of the earth around its own axis and around the sun, the earthly environment exposes its inhabitants to predictable periodic changes, most notably daily changes in lightness–darkness and food availability and seasonal changes in photoperiod. In order to adapt and anticipate to the daily changes in the environment, most organisms have evolved an internal timing system, the so-called circadian clock system. Via this system, humans and other mammals for instance lower their heart rate and body temperature to prepare themselves for sleep. This biological clock system also enables the body to switch its main metabolic substrate from carbohydrates during the active phase (daytime in humans and nighttime in nocturnal animals) to lipids during the inactive phase (nighttime in humans, daytime in nocturnal animals), to ensure adequate substrate usage by the main metabolic pathways that can provide cellular ATP.

**Figure 1:**
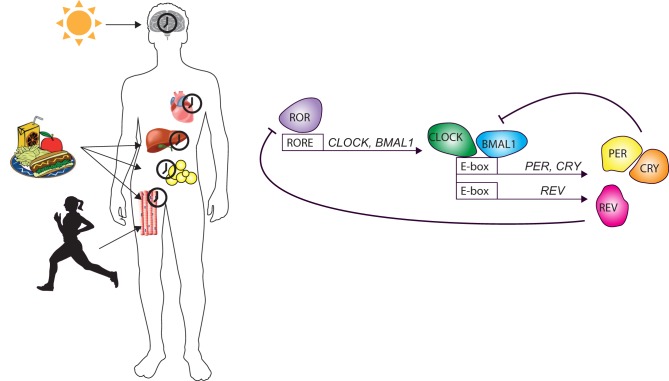
The molecular circadian clock and tissue-specific clocks in the body. CLOCK and BMAL1 form a heterodimer that binds to E-box regulatory sequences *PER/CRY* and other genes. PER and CRY form a repressor complex, which inhibits*CLOCK-BMAL1* when sufficient levels are reached. The second feedback loop involves nuclear orphan receptors, which bind to the retinoic acid-related orphan receptor response elements (ROREs) in *CLOCK* and *BMAL1* regulatory sequences. Retinoid-related orphan receptor (ROR) activates transcription of *CLOCK* and *BMAL1*. The CLOCK-BMAL1 complex induces transcription of *REVERBA* and *REVERBB* (REV), which subsequently compete with ROR, in order to inhibit transcription of *CLOCK* and *BMAL1*. Circadian clocks exist in almost every cell and exhibit tissue-specific rhythmicity, orchestrated by the central circadian clock in the suprachiasmatic nucleus. Synchronization takes place via neural, hormonal and behavioral signals.

In mammals, the circadian timekeeping system consists of a central pacemaker in the brain located in the suprachiasmatic nuclei (SCN) in the anterior hypothalamus. This central pacemaker receives input from (day) light, which it uses to synchronize its circadian rhythm of approx. 24 h with the exact 24-h daily rhythm of the rotations of the earth around its axis. Subsequently, the central pacemaker in the SCN can synchronize the different peripheral tissue clocks in the body via various signaling cascades (Fig. 1). The peripheral tissue clocks additionally receive synchronizing inputs from several other cues, such as body temperature, locomotor activity, feeding behavior and the dietary composition of food. On a molecular level, both the central and peripheral clocks use a similar mechanism that consists of a transcriptional–translational feedback loop (TTFL). The core of this TTFL consists of a negative and positive limb that ensures oscillation of the timing system, as well as several auxiliary mechanisms that allow for adjusting properties of the timing system, such as period length and flexibility or robustness of the clock mechanism (Fig. 1). The molecular circadian clock and its time keeping function are conserved in all tissues of the body, but cellular functions downstream of the molecular clock mechanism are tissue specific and only a small number of clock-controlled genes show rhythmicity in all tissue (Zhang *et al*. 2014*a*). Moreover, both the central and peripheral tissue clocks can be entrained to specific time cues (*Zeitgeber* *s*) such as light for the SCN, feeding for the liver and physical activity for skeletal muscle.

Mitochondria are often termed the cell’s powerhouses as these organelles are the main source of cellular ATP, which is produced during aerobic respiration. Within these cytosolic double-membrane organelles glucose, lipids, ketogenic and amino acid derivatives are metabolized in the tricarboxic acid cycle (TCA cycle) in order to generate H^+^ and electrons, which in turn are needed for the electron transfer chain (ETC) to produce ATP. ATP production during the aerobic ETC is highly efficient as compared to ATP production during anaerobic fermentation, gaining over tenfold higher yields (Hochachka 1993, Mitchell 1996, Rich 2003). Other, lesser known, roles of mitochondria include cellular signaling, cell growth and proliferation as well as cell death. Mitochondria contain their own (circular) DNA, mitochondrial DNA (mtDNA), as well as transcription and translation machinery and therefore can replicate independent of normal cell division. This mitochondrial replication allows for the presence of many (up to several thousand) mitochondria per cell (Cummins 2002, Cole 2016).

Recently, interest in regulation of mitochondria by the circadian timekeeping system has gained interest as more and more evidence indicates that the biological clock also orchestrates the functioning of mitochondria. This review will provide an overview of the recent findings on how the circadian clock and mitochondrial functioning are interrelated and will focus on mitochondrial morphology and mitochondrial respiration.

## Mitochondrial morphology and functioning

The energy producing capacity of mitochondria is strongly related to their abundance and morphology. In fact, mitochondrial morphology, number and functioning are highly dynamic. On a tissue level, it is well established that mitochondrial content, in terms of mtDNA, mRNA, protein content as well as enzymatic activity and respiration rates can differ up to a 100-fold between tissue types and that mitochondrial content and functioning is also species dependent (Leary *et al*. 1998, Forner *et al*. 2006, Hulbert *et al*. 2006, Fernández-Vizarra *et al*. 2011). Strikingly, a small proportion of the mitochondrial proteome seems to be unique to individual tissue types such as liver, skeletal muscle and heart (Forner *et al*. 2006, 2009). Moreover, also the substrate preference of mitochondria can differ for tissue types, e.g. glycolytic type IIa muscle fibers preferably oxidize glycolytic substrates, whereas oxidative type II muscle fibers also more readily utilize fatty acids and ketones (Forner *et al*. 2006). On a cellular level, the number of mitochondria present in an individual cell is highly variable, likely reflecting the metabolic needs of the cell. In order to suffice these highly variable changes in supply and demand of energy, cells need an efficient machinery for both mitochondrial biogenesis as well as mitophagy, the process of mitochondrial breakdown. The balance between mitochondrial biogenesis and mitophagy determines the mitochondrial content of the cell. PGC1A and PGC1B are considered to be key proteins for mitochondrial biogenesis, key proteins for mitophagy are PINK1, PARKIN and BNIP3.

**Figure 2:**
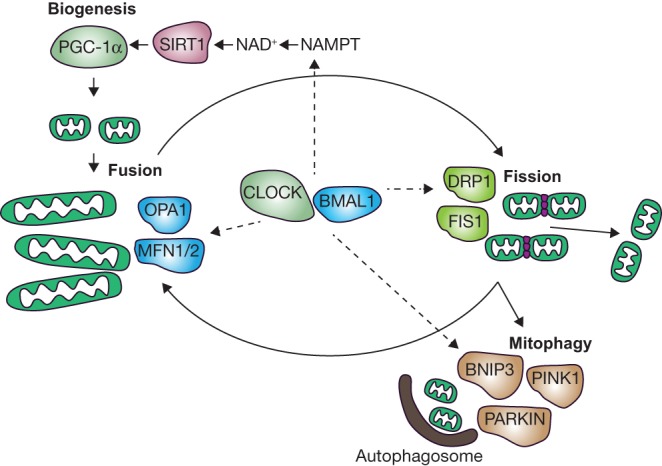
Model for circadian regulation of mitochondrial homeostasis. To maintain healthy mitochondria, mitochondria are continuously formed and removed during the active phase. The CLOCK-BMAL1 complex stimulates mitochondrial biogenesis and mitophagy through activation of SIRT1. Mitochondrial biogenesis is regulated by transcription factor PGC1A. Newly formed mitochondria fuse to form a tubular network. Mitophagy is preceded by mitochondrial fission in order to form fragmented mitochondria which can be taken up by an autophagosome. Both fusion and fission are influenced by CLOCK-BMAL1. A number of regulatory proteins regulate fusion (OPA1, MFN1/2), fission (FIS1, DRP1) and mitophagy (PINK1, BNIP1, PARKIN) processes.

Aside from the overall number of mitochondria present in a cell, the size and shape of mitochondria also plays an important role in their energy production. Mitochondria can be present not only as small individual organelles (fissioned mitochondria), but also as extensive tubular networks resulting from the fusion of multiple mitochondria (Fig. 2). These elongated, fused, mitochondria display a higher mitochondrial respiration and can be found in energy-consuming cells as well as during energy-consuming processes. Key players for mitochondrial fusion are mitofusion (MFN) 1 and 2 and optic atrophy 1 (OPA1). Key players for mitochondrial fission are dynamin-related protein 1 (DRP1) and fission 1 (FIS1) (Mitra 2013). This interplay between mitochondrial fusion and fission is known as mitochondrial dynamics.

### Biogenesis

Mitochondrial biogenesis is not simply the increase in number of mitochondria, but is often also accompanied by an increase in mitochondrial size and mass, i.e. changes in mitochondrial morphology (Jornayvaz & Shulman 2010). As the mtDNA only contains 37 genes, mitochondrial biogenesis requires the correct synthesis and import of approximately 1000–1500 proteins encoded by nuclear DNA. Overexpression of transcription factor PGC1A provided the first evidence of its role in mitochondrial biogenesis. In muscle cells, overexpression of PGC1A stimulated mtDNA copy number as well as the proliferation of mitochondria (Wu *et al.* 1999). Later *in vivo* studies indicated that expressing PGC1A in glycolytic mice muscles activated genes of mitochondrial oxidative metabolism and that inducing PGC1A expression through muscle stimulation increased mitochondrial biogenesis (Lin *et al.* 2002, Irrcher *et al*. 2003). PGC1α activity can be regulated through phosphorylation by AMPK and deacetylation by SIRT1 (Diaz & Moraes 2008). These posttranslational modifications of PGC1A likely provide a direct link between cellular nutrient status and mitochondrial biogenesis since both AMPK and SIRT1 can act as a nutrient sensors. Importantly, proteins of the PGC1 family are also involved in the regulation of metabolic processes such as gluconeogenesis, fatty acid beta-oxidation as well as oxidative phosphorylation (Lin *et al*. 2005).

### Mitophagy

Mitophagy is the regulated removal of damaged mitochondria by autophagosomes. Through mitophagy, cells keep a healthy pool of mitochondria, and also can adapt to the ever-changing metabolic demand of the cells. Additionally, mitophagy can provide the cell with nutrients from the phagocytized mitochondria during nutrient deprivation. Furthermore, mitophagy has been suggested to play a role in cell differentiation and maturation as well as pathogenesis (Youle & Narendra 2011, Ding & Yin 2012, Saito & Sadoshima 2015). Key proteins involved in mammalian mitophagy are PINK1, PARKIN and BNIP3. Although mitophagy itself is difficult to measure, several proxy methods have been employed to quantify mitophagy including key protein analysis with immunoblots and fluorescence microscopy, as well as electron microscopy using immunogold antibodies (Ding & Yin 2012).

### Mitochondrial dynamics

Mitochondrial morphology can drastically vary between cell types and tissues, and this is likely a response to metabolic cues from cells and their environment (Wai & Langer 2016). Through the process of fusion, mitochondria can form extensive networks, and conversely, through the process of fission, mitochondria can also show a strongly fragmented presence in a cell. It is thought that through fusion an exchange of material between healthy mitochondria is enabled while fission allows for separation of intact and damaged mitochondria (Ni *et al*. 2015). Both fission and fusion are strongly regulated by the cell, and disruption of mitochondrial dynamics is associated with aging and several diseases, including diabetes, and neurodegenerative diseases such as Huntington, Parkinson and Alzheimer’s disease (Chen & Chan 2009, Ni *et al*. 2015, Sebastián *et al*. 2017).

## Circadian control of mitochondrial function

### Biogenesis

Mitochondrial biogenesis is affected by many external and environmental factors, including exercise, caloric restriction, oxidative stress and cellular division, renewal and differentiation (Jornayvaz & Shulman 2010). Since the energy demand of cells fluctuates throughout the day, it is to be expected that mitochondrial abundance, morphology and/or functioning also fluctuates throughout the day. Indeed, more and more evidence indicates that mitochondria react to or maybe even anticipate the daily changes in nutrient availability that most organisms experience. Therefore, circadian control of these mitochondrial properties can be expected. Several studies investigated whether mitochondrial biogenesis, mitochondrial content, mitochondrial dynamics or mitochondrial functioning were regulated by the circadian clock (Table 1). Mitochondrial content, as measured by levels of mtDNA, protein content or mitochondrial mass was not found to be different throughout the day, neither in human skeletal muscle (van Moorsel *et al.* 2016) nor in synchronized immortalized human hepatic cells (Cela *et al.* 2016). In human muscle, the marker of mitochondrial biogenesis, PGC1A, was not found to be rhythmically expressed, but in synchronized immortalized human hepatic cells, mRNA levels of *PGC1A* were found to be rhythmic, with peak levels of expression proximal to peak levels of *BMAL1* expression. Animal models also show different results on the regulation of mitochondrial biogenesis by the circadian timing system. In mice, liver protein levels of PGC1A and PGC1B were found to be oscillating with peak levels at the onset of the active phase and in the middle of the inactive phase, respectively (Liu *et al*. 2007). We found *Pgc1* *a* mRNA to be rhythmically expressed in rat muscle, brown adipose tissue (BAT) and liver. *Pgc1* *b* was found not to be rhythmically expressed in muscle or BAT (de Goede *et al*. 2017, Oosterman *et al.* in preparation). It should be noted, however, that indirect measures of mitochondrial abundance and biogenesis such as protein and mRNA expression of *Pgc1a* and the abundance of mtDNA do not necessarily translate into mitochondrial biogenesis itself. Less indirect evidence for circadian clock control of mitochondrial content comes from several KO models in rodents and cell lines. KO of *Per2* in mouse embryonic fibroblasts did not lead to an altered number of mitochondria, as determined by fluorescent microscopy, nor to altered mtDNA copy numbers (Magnone *et al*. 2015). Global *Bmal1* KO as well as *ClockΔ19*-mutant mice showed reduced contractile muscle force and profound reductions in muscle mitochondrial volume and respiratory function, which were associated with altered expression levels of *Pgc1* *a* and *Pgc1b* (Andrews *et al.* 2010). In isolated liver mitochondria from global *Per1/2* dKO mice, the daily fluctuations in rate-limiting mitochondrial enzymes such as CPT1 and PDH and proteins involved in oxidative phosphorylation were abolished. In addition, the daily average protein content of PDH was decreased, indicating that PER proteins are involved in the regulation of key mitochondrial protein synthesis and thus possibly in mitochondrial biogenesis itself. However, no differences in daily average mtDNA copy number were found compared to wild-type mice (Neufeld-Cohen *et al.* 2016). In a liver-specific *Bmal1* KO mouse model, the mitochondrial content in liver was also unaffected (Peek *et al.* 2013), as well as liver *Pgc1a* mRNA and protein content. *Pgc1b* in contrast showed impaired rhythmicity in mice liver upon global *Clock* mutation as well as reduced mRNA levels in hepatocytes from global *Bmal1* KO mice (Zhang *et al*. 2014*b*, Gong *et al*. 2015). These differing findings between *Pgc1a* and *Pgc1b* seem to implicate that *Pgc1b*, but not *Pgc1a* levels are controlled by the positive limb of the circadian clock. Heart-specific *Bmal1* KO mice did not show altered mtDNA content in cardiac tissue, but microscopic assessment indicated a diminished number of mitochondria and altered morphology of these mitochondria (Kohsaka *et al*. 2014). Interestingly, cardiac *Pgc1a* levels were decreased in this KO model while mitochondrial protein content was also decreased. In a depletion model of *Reverba*, which is a negative mediator of the positive limb of the molecular circadian clock TTFL (Fig. 1), mice myoblasts showed decreased mitochondrial DNA levels, which is indicative of decreased mitochondrial abundance (Woldt *et al.* 2013). Moreover, PGC1A protein and mRNA expression were reduced both in cell cultures as well as in the soleus and quadriceps muscles of mice upon *Reverba* KO, whereas overexpression of *Reverba* in cell cultures increased *Pgc1a* mRNA expression. Taken together, these data indicate that *Pgc1a* and not *Pgc1b* is the main regulator of circadian rhythmicity of mitochondrial biogenesis as only impairment of *Pgc1a* leads to reduced mitochondrial abundance and differences in mitochondrial volume and protein content. Additionally, these data suggest that, with the exception of cardiac tissue, *Pgc1a* transcription is not affected by the positive limb of the TTFL, but disruption of the negative limb does decreases PGC1A protein expression and mitochondrial content. A possible connection between the TTFL and PGC1A are nutrient sensors such as AMPK and SIRT1, since it has been shown that REVERBA increased PGC1A activity through AMPK-dependent activation of *Sirt1* (Woldt *et al.* 2013). Conversely, PGC1A has been shown to affect the functioning of the core clock machinery by increasing transcription of *Bmal1* and *Clock* via interactions with RORa and RORb (Liu *et al*. 2007). Moreover, depletion of PGC1A impaired circadian rhythms of activity, body temperature and metabolic rate in mice, indicating that *Pgc1a* affects not only the molecular clock, but also the behavioral rhythms ultimately.

**Table 1:** Overview of findings on mitochondrial rhythms.

Species	KO tissue	KO gene	Main findings	In tissue	Ref.
Human			NR mtDNA NR mt protein NR mt mass NR PGC1A ~PINK1 and FIS1 ~OCR	Muscle	van Moorsel *et al.* (2016)
Human (cells)			~*PGC1A* ~Gluthathione	Hepatic (HepG2)	Cela *et al*. (2016)
Mouse			~PGC1A ~PGC1B	Liver	Liu *et al*. (2007)
Rat			~*Pgc1* *a*NR* Pgc1b*	Muscle and BAT	de Goede *et al*. (2017)
Rat			~*Pgc1a*	Liver	de Goede *et al*. (2017)
Mouse			~mt dynamics ~mt membrane potential ~Phagocytic/bactericidal activity	Macrophages	Oliva-Ramírez *et al*. (2014)
Rat			~OCR	Brain	Simon *et al*. (2003)
Mouse			~Several ETC mRNAs	SCN, but not liver	Panda *et al*. (2002)
Mouse			~PRXIII	Adrenal gland, BAT and heart	Kil *et al*. (2015)
Mouse			~PRXI	Liver	Edgar *et al*. (2012)
Mouse	Global	*Pgc1a* KO	NR TCA/ETC gene expression	Liver and muscle	Liu *et al*. (2007)
Mouse	Global	*Per2* KO	– mt abundance – mtDNA ↑ resistance to ROS & UV cytotoxity ↑ NADH/NAD^+^	Embryonic fibroblast	Magnone *et al*. (2015)
Mouse	Global	*Bmal1* KO and *ΔClock*	↓ muscle force, mt volume, OCR NR *Pgc1a*, *Pgc1b*	Muscle	Andrews *et al*. (2010)
Mouse	Global	*Per1/2* dKO	NR mt rate-limiting proteins↓ OCR	Liver	Neufeld-Cohen *et al*. (2016)
Mouse	Global	*Bmal1* KO	↓ *Pgc1b*	Primary mouse hepatocyte	Zhang *et al*. (2014*b*)
Mouse	Global	*ΔClock*	NR several mt genes – *Pgc1* *a*NR* Pgc1b* ↓ and NR SIRT3 NR OPA1 NR mt oxidative stress markers NR SOD acetylation and activity	Liver	Gong *et al*. (2015)
Mouse	Global	*Reverba* KO	↓ mtDNA ↓ mt abundance mt morphology altered ↓ *Pgc1a*/PGC1A ↓ ETC gene and protein expression ↓ respiration	Muscle	Woldt *et al*. (2013)
Mouse	Global	*ΔClock*	↓ ATP synthase complex proteins	Muscle	McCarthy *et al*. (2007)
Mouse	Global	*Cry1/2* dKO	↑ mt reserve capacity ↑ exercise performance	Myotubes	Jordan *et al*. (2017)
Mouse	Global	*Bmal1* KO	NR NADH levels	Epidermal stem cells	Stringari *et al*. (2015)
Mouse	Global	*Bmal1* KO	↓ mt proton gradient ↓ ATP/ADP	Pancreas (β-cells)	Lee *et al*. (2011)
Mouse	Liver	*Bmal1* KO	– mtDNA – mt biogenesis enzymes ↓ OCR ↑ *Atp5b*	Liver	Peek *et al*. (2013)
Mouse	Liver	*Bmal1* KO	NR mt Biogenesis mRNAs NR mt morphology ↑ mt size/surface NR several fission/mitophagy mRNAs – *Mfn1 Mfn2 Opa1* ↓ several fission/mitophagy proteins ↑ MFN1 ↓ OCR ↑ superoxide levels	Liver/hepatocyes	Jacobi *et al*. (2015)
Mouse	Liver (cells)	*Cry1, Cry2, Per1* or *Per2* siRNA	↑ OCR	Hepa 1–6 cell line	Jacobi *et al*. (2015)
Human (cells)	HEPG2	*BMAL1* KO	↓ mt respiration	Hepatic (HepG2)	Scrima *et al*. (2016)
Mouse	Muscle	*Bmal1* KO	↓ and NR PDH activity	Muscle	Dyar *et al.* (2014)
Mouse	Cardiac	*Bmal1* KO	↓ mt protein ↓ mt abundance – mtDNAmt morphology altered ↓ ETC gene expression and activity ↓ *Pgc1a* ↓ *Mfn1* and *Opa1* ↓ NAD^+^ and NADH	Cardiac	Kohsaka *et al*. (2014)
Mouse	Cardiomyocyte	*ΔClock*	– mtDNA, mt number, mt density – mt morphology ↓ OCR (subsarcolemmal fraction) – OCR (intra myofibrillar)	Heart (subsarcolemmal and intra myofibrillar)	Bray *et al*. (2008)
Mouse	Pancreas (β-cell)	*Bmal1* KO	↓ mt membrane potential gradient ↓ ATP/ADP ↑ ROS accumulation	Pancreas (β-cells)	Lee *et al*. (2011)

For KO studies, findings are represented as differences compared to wt animals.

–, no changes; ~, rhythmic (i.e. with at least 2 time points); ΔClock, ΔClock19 mice; dKO, double knockout; KO, knockout; mt, mitochondrial; NR, non-rhythmic or altered/dampened rhythms.

### Mitophagy

Seemingly contradictory, *Pgc1a* levels fluctuated throughout the day, but mitochondrial mass or content was not found to be fluctuating throughout the day, but did seem to be affected by disturbing the molecular clock in most of the animal and human (cell) studies described earlier. In a different study in mice liver, mitochondrial biogenesis was found to be diurnally regulated in a *Bmal1*-dependent manner, as liver-specific *Bmal1* KO eliminated the diurnal pattern of mitochondrial biogenesis (Jacobi *et al.* 2015). If mitochondrial biogenesis is indeed fluctuating while mitochondrial content does not change, as the previously mentioned indirect markers of biogenesis suggest, then the process of mitochondrial removal should act as a counter-mechanism to maintain mitochondrial homeostasis. Mitochondria are removed from the cytosol of the cell via mitochondrial-specific autophagy, called mitophagy. For autophagy, it has been shown that the number of autophagic vacuoles vary throughout the day in various tissue types (Pfeifer & Scheller 1975, Pfeifer 1981, Ma *et al*. 2012). Additionally, liver-specific knockout of *Bmal1* abolished diurnal regulation of *Bnip3* and diminished the levels of autophagy markers as well as the flux in autophagy itself (Ma *et al*. 2011). However, time of day dependence of mitophagy, has been studied less. Mitophagy and the morphology of the mitochondria are inherently linked with each other as mitochondrial fission is required for mitophagy and apoptosis (Twig *et al.* 2008). Elongated and fused mitochondria are protected from mitophagy, possibly due to their extended size, often forming tubular networks that simply do not fit into the autophagosomes (Gomes *et al*. 2011, Rambold *et al*. 2011).

### Mitochondrial dynamics

As mentioned before, key players for mitophagy are PINK1, PARKIN as well as mitochondrial autophagy receptors such as BNIP3; for mitochondrial fusion, the key proteins are considered to be MFN1 and MFN2 and OPA1, while key players for mitochondrial fission are DRP1 and FIS1 (Gomes & Scorrano 2013, Mitra 2013). Mitophagy seems to have evolved as a key mechanism for keeping a healthy pool of mitochondria in the cell, eliminating excessive/superfluous and damaged mitochondria (Gomes & Scorrano 2013). First evidence of timely regulation of fission, fusion and mitophagy came from BMAL1 ChIP-seq experiments showing that mediators of fission such as *Fis1*, as well as mitophagy regulators such as *Pink1* and *Bnip3* were direct targets of BMAL1 and their expression levels were also found to be affected upon liver-specific *Bmal1* KO (Rey *et al*. 2011, Koike *et al*. 2012, Jacobi *et al.* 2015). Fission-related protein DRP1 and regulators of fusion MFN1/2 and OPA1 were not found to be direct targets of the hepatic molecular clock (Jacobi *et al.* 2015). In contrast, in cardiac tissue fusion-related *Mfn1* and *Opa1*, mRNA expression was downregulated in heart-specific *Bmal1* KO mice, suggesting that Bmal1 indirectly affects mitochondrial fusion in the heart (Kohsaka *et al*. 2014). In human skeletal muscle and mice liver, PINK1 protein levels showed opposite acrophases as compared to FIS1, suggesting that these processes do not take place at the same time (Jacobi *et al.* 2015, van Moorsel *et al.* 2016). In human muscle, PINK1 protein levels peaked in the middle of the active phase, while in mouse liver, PINK1 peaked at the end of the active phase. Confocal microscope morphology studies showed that in synchronized murine macrophages, the mitochondria follow a rhythmic pattern in their fission/fusion dynamics and that a more fused state correlated with an increased membrane potential of mitochondria as well as increased macrophage phagocytic and bactericidal activity (Oliva-Ramírez *et al*. 2014). This suggests that mitochondria prepare themselves for high energy demanding activity such as during the active phase through the process of mitochondrial fusion. Nevertheless, these results could not be reproduced in an *ex vivo* setting, as no diurnal differences were found in membrane potential nor phagocytic capacity of the macrophages when using freshly isolated macrophages (Oliva-Ramírez *et al*. 2014). These findings suggest that mitophagy takes mainly place during the active phase and that both fission and mitophagy are regulated by the positive limb of the TTFL. Another possible regulator of mitophagy is SIRT1. Addition of nicotinamide, a precursor of NAD^+^, increased mitophagy in human fibroblasts and this was dependent on the NAD^+^ sensor SIRT1 (Kang & Hwang 2009, Jang *et al*. 2012). Interestingly, SIRT1 target PGC1A levels peak during the active period (Liu *et al*. 2007, Diaz & Moraes 2008). Taken together, these findings seem to suggest that both mitochondrial biogenesis and mitophagy are enhanced during the active phase and that both processes are perhaps regulated via SIRT1. Mitochondrial quality control thus seems to be regulated by the circadian clock, either via BMAL1 or more indirectly via SIRT1, leading to increased turnover of mitochondrial content during the active phase, but without clear alterations in mitochondrial content throughout the day. However, further studies on these subjects are desired, as most studies on mitochondrial biogenesis, mitophagy and mitochondrial dynamics do not directly measure these processes, but instead rely on biomarkers of these processes.

## Circadian control of mitochondrial respiration

Energy requirements of all organs are dependent on their activity levels. Furthermore, substrate usage can differ between carbohydrate and fat oxidation in the active and inactive phase. Thus, it would be efficient to prepare mitochondrial respiratory capacity according to the light and darkness phase. Since expression of clock-controlled genes exhibits high organ specificity and peripheral oscillators use individual entrainment signals (i.e., feeding and physical activity), we summarize the key literature findings separately for the different organs (Table 1).

An important confounder in the investigation of clock-controlled mitochondrial processes is the influence of pervasive external factors also showing daily rhythmicity, most notably nutrient availability. Most processes *in vivo* are not only controlled by the biological clock, but also by other factors such as feeding and activity state and the light–darkness cycle. To investigate the contribution of the biological clock specifically, it is necessary to tease apart the influence of the biological clock from all behavior-related influences. Over the last decades, a number of techniques have become available that make it feasible to study the biological clock at the system and organ level. In rodent models, genetic disruption of the biological clock allows to study the isolated influence of a disturbed clock on mitochondrial processes. Frequently used models in this research comprise the disruption of the TTFL, mainly targeting *Bmal1, Clock, Per2* and *Cry1*. Alternatively, disrupting natural feeding–fasting cycles by using time-restricted feeding paradigms have been applied in many different forms and demonstrated to influence biological rhythms of different organs (Hatori & Panda 2015). Furthermore, keeping animals fasted and in constant dark conditions before collecting the tissues of interest prevents the acute influence of light and feeding (Peek *et al*. 2015). In addition, *in vitro* studies can be used to study cells that are isolated of potentially confounding rhythmic influences, such as feeding or neuronal and hormonal signals. After synchronizing the cells by e.g. serum shock, circadian rhythms of the TTFL and downstream processes are largely maintained and can be measured at several time points. As a downside, *in vitro* studies do not allow to study the complex regulation of the biological clock on the organ or system level.

### Brain

One of the earliest observations indicating that mitochondrial function is variable over the day comes from studies in the brain. Mitochondrial oxygen consumption, measured in isolated mitochondria from whole-brain homogenates, was highest in the middle of the resting phase. This was either in the absence of ADP (state 4 respiration) or upon ADP stimulation (state 3 respiration) (Simon *et al*. 2003). Furthermore, the respiratory control ratio (RCR; state 3/state 4), a measure of how efficient mitochondrial respiration is coupled to ATP production, showed diurnal differences. In the SCN of mice, over 300 genes exhibit rhythmic expression over 24 h (Panda *et al*. 2002). Interestingly, several of these genes code for components of the ETC in mitochondria and peak toward the end of the light phase. These results suggest influence of the molecular clock over energy-providing processes in SCN neurons to match increased metabolic demand to the higher activity in the light phase, i.e. both in nocturnal and diurnal species, highest firing rates in SCN neurons are observed during the light period (Sato & Kawamura 1984, Meijer *et al*. 1998, Nakamura *et al*. 2008).

### Liver

A large part of the hepatic transcriptome, proteome and metabolome exhibits tissue-specific circadian rhythmicity and has been studied extensively in cell and animal models. Therefore, it is not surprising that important functions of cellular energy metabolism, such as mitochondrial respiration are under circadian control. Disrupting *Bmal1* transcription by siRNA in a liver-derived cell line (HepG2) led to a decrease in mitochondrial respiration (Scrima *et al.* 2016). In mice, genetic ablation of *Bmal1* at the whole-body level resulted in a decreased oxygen consumption rate (OCR) in isolated liver mitochondria (Peek *et al.* 2013). Interestingly, OCR upon addition of a fatty acid substrate was lower at both the end and the beginning of the subjective darkness (i.e. active) phase in this study, suggesting overall lower mitochondrial respiration upon circadian disturbance. Further, the same study identified that specifically beta-oxidation and the TCA cycle, catabolic steps upstream of the ETC, caused the decrease in respiration (Peek *et al.* 2013). In support of these findings, a liver-specific knockdown of *Bmal1* also resulted in lower OCR in response to a glycolytic substrate of mitochondria in both the active and inactive phase of mice (Jacobi *et al.* 2015). These results indicate that the peripheral molecular clock exerts control over mitochondrial respiration. In addition to the influence of BMAL1 on mitochondrial respiration also PER1/2, negative elements of the TTFL, seem to be important regulators. In mice, ablation on the whole-body level of *Per1* and *Per2* resulted in a decrease in mitochondrial OCR over the day when using either a fatty acid or glycolytic substrate (Neufeld-Cohen *et al.* 2016). In stark contrast, abrogation of the transcription of *Per1* or *Per2* in the Hepa 1–6 cell line, resulted in increased mitochondrial OCR (Jacobi *et al.* 2015). It could be speculated that the differences in the model (i.e. cell line vs *in vivo* model) may contribute to the contradicting effect on mitochondrial respiration. However, an explanation of the mechanism that leads to this different response is missing.

**Figure 3:**
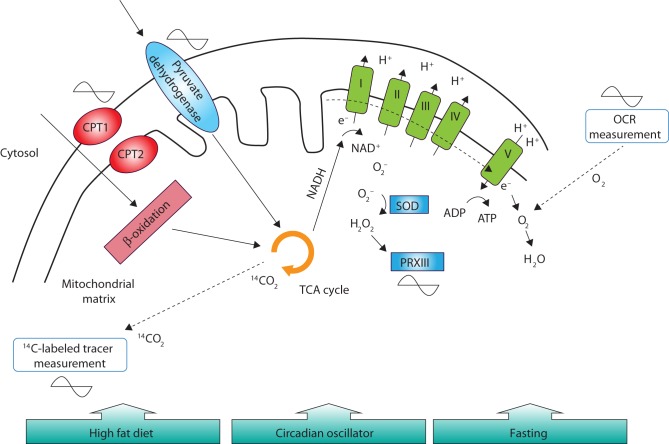
Mitochondrial respiration and ROS production show rhythmic activity. Mitochondrial respiration is the result of electron transfer to molecular oxygen as final step in the electron transport chain (ETC). Respiration is experimentally determined by measuring OCR. In addition, ^14^C-labeled substrates can be used to assess mitochondrial energy production by measuring ^14^CO_2_. Substrate transport (CPT1/2, PDH) and catabolic processes (β-oxidation, TCA cycle) also exhibit circadian rhythms and are under control of the circadian clock. Consequently, mitochondrial respiration is rhythmic in various cell and animal models. Mitochondria are also a source of ROS that are produced in various sites, such as complex I of the ETC. Superoxides (O_2_^−^) are scavenged by superoxidedismutase (SOD) and reduced to H_2_O_2_. Several antioxidant proteins such as peroxiredoxins (PRXIII) subsequently eliminate H_2_O_2_. Also antioxidant proteins and ROS production display circadian activity. Additionally, feeding behavior, including diet composition, also affects mitochondrial processes.

While these studies show that an intact circadian clock is necessary for normal mitochondrial respiration, it has also been shown that respiration exhibits an intrinsic circadian rhythm. Mitochondrial OCR in response to a glycolytic substrate in isolated hepatocytes from mice was shown to be higher near the end of the active phase (Jacobi *et al.* 2015). In liver tissue from fasted mice, ^14^C-labeled substrate oxidation exhibited robust 24-h oscillations with a peak toward the end of the resting phase (Peek *et al.* 2013). Intriguingly, when using substrates that require the glycolytic pathway (pyruvate), OCR was shown to peak at the beginning of the inactive period, whereas OCR upon fatty acid substrates (palmitoyl CoA) peaked 8 h earlier at the end of the active period (Neufeld-Cohen *et al.* 2016). This difference in peak respiration might be due to the rate-limiting enzymes of the respective pathways. Protein levels of pyruvate dehydrogenase (PDH, rate limiting for the glycolytic pathway) and palmitoyl-transferase 1 (CPT1, rate limiting for the fatty acid pathway) displayed a rhythm that corresponded to the rhythm of OCR upon the same substrate and was dependent on normal PER1/2 function (Fig. 3) (Neufeld-Cohen *et al.* 2016). While these studies uniformly show diurnal changes in mitochondrial respiration, the time of peak respiration varies clearly. Since these studies were performed with either whole tissue, cells or isolated mitochondria, it could be speculated that the extra-mitochondrial and extra-cellular environment determines the difference in time keeping. Moreover, external influences, such as nutrients and feeding/fasting, seem to exert additional control over circadian rhythms of respiration. Mice on a high-fat diet (HFD) lose rhythmicity in respiration, which highlights the vulnerability of the coordinated mitochondrial function by the circadian clock (Neufeld-Cohen *et al.* 2016). Moreover, hepatocytes from fasted mice display no difference in OCR over the day (Jacobi *et al.* 2015). However, fasting might affect specifically mitochondrial respiration at the level of the ETC, as ^14^C-labeled substrate oxidation remains rhythmic upon fasting condition (Peek *et al.* 2013).

Together, these studies indicate that hepatic mitochondrial respiration is influenced by the circadian clock, but it remains to be answered, which steps exactly in the pathway of substrate oxidation are under circadian control and how the molecular circadian clock regulates these pathways.

### Muscle

Physical activity patterns in animals under laboratory conditions exhibit clear circadian rhythmicity, which is abrogated upon disruption of the circadian clock (Mosko & Moore 1979, McDearmon *et al.* 2006). Furthermore, human studies show that exercise performance is variable throughout the day (Conroy & O’Brien 1974, Facer-Childs & Brandstaetter 2015). To regulate bio-energetic demand in skeletal muscle, the circadian clock might impose control over important metabolic processes such as mitochondrial respiration. Disrupting the circadian clock by muscle-specific *Bmal1* ablation resulted in a substantial decrease in ^14^C-glucose oxidation in isolated mouse diaphragms (Dyar *et al.* 2014). While this decrease in glucose oxidation could be due to impaired respiration (which was not assessed separately in this study), activity of the rate-limiting enzyme pyruvate dehydrogenase (PDH), upstream of respiration, was lower over a 24-h period. This suggests that, similar to liver mitochondria, important catabolic enzymes could determine fluctuations in mitochondrial respiration. A dysfunctional circadian clock, induced by whole-body Bmal1 abrogation, also directly impairs respiration. Thus, mitochondrial oxygen consumption under ADP-titration (state 3 respiration) was decreased markedly in gastrocnemius and diaphragm muscle of *Bmal1* KO mice, resulting in a decrease in RCR (Andrews *et al.* 2010). Similarly, whole-body knock down of the accessory clock component *Reverba* in mice resulted in decreased state 3 respiration in isolated mitochondria and in permeabilized muscle fibers of the soleus muscle (Woldt *et al.* 2013). In addition, protein levels of respiratory chain complexes 1, 3 and 4 were strongly decreased. Interestingly, overexpressing *Reverba* improved *ex vivo* mitochondrial respiration (Woldt *et al.* 2013). Impairments of components of the ETC might also occur due to a defective CLOCK protein. At least on the transcriptional level, a dominant-negative *Clock* mutation resulted in a decrease in expression levels of several subunits of the ATP synthase (McCarthy *et al*. 2007). Of note, a recent study found that deletion of *Cry1* and *Cry2*, negative regulators of BMAL1/CLOCK, resulted in increased mitochondrial reserve capacity in primary myotubes and increased exercise performance in mice (Jordan *et al.* 2017). This effect was possibly mediated by PPARD, since CRY1/2 exerts a repressor function under normal conditions. Together, these studies highlight that mitochondrial respiration in skeletal muscle is dependent on the molecular circadian clock machinery. However, also in muscle, the exact properties of this regulation, remain to be investigated.

Whether mitochondrial respiration exhibits circadian rhythmicity has been investigated in a limited number of studies. Cell lines in culture can display robust circadian oscillations after they are synchronized by an overwriting signal, e.g. after being exposed to serum shock (Peek *et al*. 2015). Accordingly, mouse skeletal muscle derived C2C12 myotubes show circadian rhythmicity (monitored over 48 h) in oxidation of ^14^C-labeled fatty acids (Peek *et al.* 2013). Interestingly, ^14^C-labeled glucose as substrate resulted in a similar rhythmic oxidation, which was shifted by 4 h. In the same study, also OCR, a direct measurement of mitochondrial respiration, showed a circadian rhythm over 48 h. While this suggests direct control of the circadian clock on mitochondrial respiration in myocytes, it should be noted that crucial components of upstream processes, such as in beta-oxidation and glycolysis, also exhibit circadian rhythmicity (Hodge *et al*. 2015). This adds another layer of complexity to the circadian regulation of substrate utilization. A recent study from our lab reported daily fluctuations in mitochondrial respiration in human skeletal muscle tissue, measured in permeabilized muscle fibers from muscle biopsies (van Moorsel *et al.* 2016). OCRs during APD-stimulated state 3 respiration showed pronounced diurnal changes with peak and trough at 23:00 h and 13:00 h, respectively. Mitochondrial respiration was assessed using fatty acids, glutamate and succinate as substrates. Interestingly, at 23:00 h *BMAL1* also exhibited peak expression levels, while *PER2* expression was at its trough (van Moorsel *et al.* 2016).

Taken together, ample evidence indicates that mitochondrial oxidative metabolism in skeletal muscle is under control of the peripheral circadian clock.

### Other tissues

The circadian control of mitochondrial respiration has also been shown in other tissues such as the heart, beta-cells and epidermal stem cells. In the heart of *Clock^∆19^*-deficient mice, the subsarcolemmal fraction of mitochondria showed a decreased state 3 OCR, while intramyofibrillar mitochondria were not affected (Bray *et al.* 2008). Another study demonstrated that heart-specific *Bmal1* knockdown in mice is associated with downregulated expression levels of genes belonging to the TCA cycle and ETC, together with reduced complex I activity (Kohsaka *et al*. 2014). In a model of epidermal stem cell imaging, the amount of free NADH was used as a proxy marker for oxidative metabolism and showed fluctuations with a circadian pattern. These fluctuations were not present in cells derived from *Bmal1*-deficient mice (Stringari *et al*. 2015). Another potential implication for clock-controlled mitochondrial respiration was reported in a study using insulin producing beta-cells. Here, deleting *Bmal1* resulted in an increase in uncoupling protein 2 (UCP2), which resulted in a decreased inner mitochondrial membrane proton gradient and thus to a decrease in the ATP/ADP ratio (Lee *et al*. 2011). Since the ATP/ADP ratio is an important cue for insulin secretion, this observation may link the molecular circadian clock machinery via mitochondrial respiration to insulin secretion.

## Mitochondrial redox homeostasis and the circadian clock

Mitochondrial respiration is connected to production of reactive oxygen species (ROS) and mitochondria are major ROS production sites in the cell (Sena & Chandel 2012). While the perils of ROS have been thoroughly debated in the past, attention has more recently shifted toward the physiological necessity of ROS to maintain cellular viability. Importantly, ROS play a major role as signaling molecules that regulate various crucial cellular processes, such as autophagy, immunity, differentiation and response to hypoxia (Sena & Chandel 2012). Disturbance of redox homeostasis can impair important signaling events, which can result in cell damage, making it necessary to tightly regulate ROS production and removal. Several proteins are involved in elimination of ROS in the mitochondria and cytosol. Notable antioxidant proteins include catalase, glutathione, thioredoxin and peroxiredoxin (Reczek & Chandel 2015). In addition, uncoupling proteins in the mitochondrial membrane may alleviate ROS production (Mailloux & Harper 2011).

Keeping redox homeostasis in balance depends on ROS production and ROS scavenging. Generation of ROS in mitochondria occurs when electrons are occasionally transferred to oxygen (O_2_), generating a superoxide molecule (O_2_^−^) (Fig. 3). Superoxides are eliminated by catalyzing them into hydrogen peroxide (H_2_O_2_) by the enzyme superoxide dismutase (SOD). Generation of superoxides occurs at several sites, but complexes of the ETC are a major source (Mailloux 2015). Production rates of mitochondrial superoxides are mainly determined by the redox state of electron carriers (i.e. ratio of NADH/NAD^+^) and the inner mitochondrial membrane proton gradient (Sena & Chandel 2012). An efficient way to alleviate superoxide production might be the dissipation of the proton gradient by uncoupling proteins (Mailloux & Harper 2011). Interestingly, expression levels of *Ucp3* in rat heart exhibit diurnal variations with highest levels in the resting phase (Stavinoha *et al*. 2004). Moreover, measuring ROS production in a model of synchronized HepG2 cells revealed highest levels at the time of peak OCR (Cela *et al.* 2016). Since it is methodologically cumbersome to directly assess superoxide production, most studies focus on measuring antioxidant status (Mailloux 2015). A study in athletes found that plasma levels of glutathione and catalase are higher in the evening compared to the morning, suggesting higher capacity to cope with oxidative stress (Ammar *et al.* 2015). Mechanistic studies in mouse MEFs revealed increased catalase levels upon constitutive overexpression of *Reverba*, suggesting a direct link to the molecular clock (Sengupta *et al.* 2016). In a study of mouse liver, glutathione expression was rhythmic and peaked at the beginning of the active phase (Xu *et al*. 2012). In addition, the mitochondrial isoform of peroxiredoxin (PRXIII) in mice peaks at the end of the active phase in adrenal gland, brown adipose tissue and heart (Kil *et al*. 2015). Similarly, in mouse liver tissue, the cytosolic and nuclear isoform PRXI was reported to be higher toward the end of the active phase (Edgar *et al.* 2012). Together, these studies suggest that ROS production occurs during the active phase.

Another important regulation of ROS production might be facilitated through fusion and fission processes. In primary hepatocytes from *Bmal1*-depleted mouse liver, superoxide levels were increased and mitochondria were swollen. Upon induction of FIS1, a mitochondrial fission-promoting protein, superoxide levels were decreased, suggesting that BMAL1 can influence ROS production through morphological changes of mitochondria, which is in line with the effects of fission on mitochondrial respiration described earlier (Jacobi *et al.* 2015).

Recent evidence shows that the mitochondrial redox system is linked to the biological clock through the NAD^+^-dependent deacetylase SIRT1. The cytoplasmic and nuclear enzyme SIRT1 is activated in response to varying NAD^+^ levels and causes deacetylation of among others *BMAL1* in a rhythmic manner (Nakahata *et al*. 2008). In addition, SIRT1 has also been shown to deacetylate PER2, reducing its activity and affecting the circadian rhythmicity of core clock genes (Asher *et al*. 2008). It is important to emphasize that SIRT1 also regulates the activity of PGC1A, an important activator of mitochondrial biogenesis (Rodgers *et al*. 2005). There also appears to be indirect regulation of redox metabolism by the biological clock through the NAMPT–NAD^+^–SIRT3 axis. The rate-limiting enzyme in the NAD^+^ salvage pathway, NAMPT, is controlled by the core molecular clock (Nakahata *et al*. 2009, Ramsey *et al.* 2009). Another pathway involves the nicotinamide riboside (NR) pathway, in which key enzymes of NAD^+^ synthesis (i.e. NRK1 and NMNAT) are under clock gene control (Mauvoisin *et al.* 2017). In agreement, NAD^+^ levels in cell and animal models have been shown to fluctuate with core molecular clock oscillations (Nakahata *et al*. 2009, Ramsey *et al.* 2009). The activity of the mitochondrial deacytelase SIRT3 is NAD^+^ dependent and has important regulatory functions for mitochondria proteins. Importantly, the acetylation status of mitochondrial proteins also shows a clear temporal separation. In a recent study, SIRT3-targeted proteins in mouse liver were found predominantly acetylated during the resting phase of the animals (Mauvoisin *et al.* 2017). Several proteins of redox homeostasis in mitochondria are under control of SIRT3. Among these regulated proteins is mitochondrial SOD2, which catalyzes the initial reduction of superoxide into H_2_O_2_ and exhibits rhythmic acetylation and activity in mouse liver. In mice with the *Clock^Δ19^* mutation, this rhythm in SOD2 acetylation and activity is abrogated (Gong *et al*. 2015). In addition to changes in NAD^+^ by NAMPT, the balance of NADH/NAD^+^ can also be regulated through changes in NADH levels. Since NADH is oxidized to NAD^+^ by complex I of the ETC, a decrease in its activity results in a higher NADH/NAD^+^ ratio. Mutations in *Per2* led to a decreased complex I activity and to a higher NADH/NAD+ ratio, indicating that the molecular circadian clock has multiple ways to adjust the redox balance (Magnone *et al*. 2015). This is of importance, since NADH is required for the efficient binding of the heterodimer BMAL1/CLOCK (Rutter *et al*. 2001). Adding to this, recent evidence showed that NADH levels exhibit circadian oscillations (Huang *et al*. 2016). It should, however, be noted that the NAD*^+^*/NADH ratio has multiple crucial functions in mitochondrial homeostasis that extend far beyond its involvement in ROS production.

In order to convey information to other cytosolic compartments, redox metabolites from the mitochondria, such as H_2_O_2_, must be transported into the cytosol. An intricate regulatory system, which shows circadian activity, has evolved to facilitate this transport. In mitochondria, PRXIII is the main scavenger for H_2_O_2_ and gets oxidized. Subsequently, PRXIII can be recycled by another enzyme, thioredoxin. High H_2_O_2_ levels lead to overoxidation of PRXIII, protecting it from being recycled (Rhee & Kil 2016). In a study of mouse BAT, lung and heart, overoxidized PRXIII peaks at the end of the active phase (Kil *et al*. 2015). Recently, a model of circadian regulation of H_2_O_2_ signaling has been proposed in which PRXIII overoxidation by high H_2_O_2_ levels leads to spillover of H_2_O_2_ from mitochondria into the cytosol. Once H_2_O_2_ is in the cytosol, it can exert several functions, such as activating the mitogen-activated protein kinase (MAPK) signaling pathway or decreasing steroidogenesis in the adrenal gland (Kil *et al*. 2012, Rhee & Kil 2016). Interestingly, H_2_O_2_ in the cytosol might also act as starting signal for a negative feedback loop, which leads to complete recycling of PRXIII in the mitochondria and thus to abrogation of H_2_O_2_ transport into the cytosol (Rhee & Kil 2016). The latter mechanism has been postulated to regulate corticosterone production in a diurnal fashion in mice, in addition to the well-known input from the HPA axis (Kil *et al*. 2012).

Taken together, accumulating evidence indicates that mitochondrial ROS production and scavenging shows similar diurnal fluctuations as mitochondrial oxidative phosphorylation. In addition to diurnal ROS variation due to circadian regulation of oxidative phosphorylation, it appears that the molecular circadian clock has direct links to important regulatory steps of ROS production and scavenging. An intriguing finding is the discovery of a secondary feedback loop which links mitochondrial H_2_O_2_ production to intracellular signaling.

## Conclusion

More and more evidence indicates that the circadian clock and mitochondrial functioning are related. Most available evidence shows how the circadian clock controls the abundance and morphology of mitochondria by regulating biogenesis, fission/fusion and mitophagy. Additionally, several studies suggest that mitochondrial functioning also is regulated by the circadian clock as KO studies show altered mitochondrial respiration and ROS metabolism, although in these studies, it is difficult to separate effects on substrate availability and mitochondrial function itself. Conversely, direct evidence for mitochondrial regulation of feedback to the circadian clockwork is very limited.

However, for a better understanding of how mitochondrial morphology and functioning change throughout the day, more experiments are needed. Performing imaging and respiration assays throughout the day in different tissues seems to be essential in order to get a clearer picture whether morphology and respiration oscillate throughout the day, whether this is tissue dependent and whether this is related to the molecular clock, substrate availability or a combination of both. Furthermore, data on other regulators such as hormone signaling and the autonomic nervous system, both outputs of the central clock, are scarce, but potentially also exert influences on mitochondrial functioning. One first candidate hormone to investigate is melatonin, which for a long time has been known to be both a hormonal output of the central clock as well as an antioxidant.

## Declaration of interest

The authors declare that there is no conflict of interest that could be perceived as prejudicing the impartiality of this review.

## Funding

This work was supported by a grant from the Netherlands Organisation for Scientific Research (NWO; TOP grant number 40-00812-98-14047, 2015).
